# Shedding light on the brain: guidelines to address inconsistent data collection parameters in resting-state NIRS studies

**DOI:** 10.3389/fnins.2025.1557471

**Published:** 2025-04-09

**Authors:** Nick W. Bray, Abby Blaney, Michelle Ploughman

**Affiliations:** Recovery and Performance Lab, Biomedical Sciences, Faculty of Medicine, Memorial University of Newfoundland, St. John's, NL, Canada

**Keywords:** methods, experimental design, data analysis, functional neuroimaging, optical tomography, diffuse optics, older adult, dementia

## 1 Introduction

Butters and colleagues, in their recent and eloquent review, suggest that near-infrared spectroscopy (NIRS) is a promising tool for exploring brain function across the dementia spectrum (Butters et al., 2023); dementia is a clinical syndrome affecting memory and thinking skills, but we still do not entirely understand the underlying pathophysiology, given the absence of a pharmaceutical cure and the numerous culprits (i.e., protein accumulation, neuronal death, vascular injuries, etc.; Hachinski, 2019; Alzheimer's Association, 2024). The number of studies using NIRS in dementia has increased from ~5 per year in 2006 to just over 85 in 2023 (Butters et al., 2023). In brief, NIRS is a non-invasive neuroimaging technique that uses light within the near-infrared range (i.e., ~650–1,000 nanometers; Jobsis, 1977) to monitor brain oxygenation and, by extension, brain activity as a result of the hemodynamic response (Ogawa et al., 1990).

In their review, Butters and colleagues examined 88 NIRS studies, including 32 collecting resting-state data and 65 employing a task-based paradigm; several studies acquired data under both conditions (Butters et al., 2023). Broadly, a task-based (i.e., extrinsic activity) condition requires an individual to perform a cognitive and/or physical function test, such as pressing a button in response to a stimulus (Knutson et al., 2000; Linden et al., 1999). Conversely, a resting-state (i.e., intrinsic activity) condition requires an individual to remain stationary and engage in unconstrained mental activity, such as “daydreaming” or “mind wandering” (Biswal et al., 1995; Mason et al., 2007). Each condition provides complementary insight into brain function, and both have been collected as part of cross-sectional (Milham et al., 2002; Bray et al., 2023a), longitudinal (McLaren et al., 2012; Damoiseaux et al., 2012), and interventional (Liu-Ambrose et al., 2012; Bray et al., 2023b) research. However, resting-state NIRS may serve as an indicator of baseline brain activity, as it captures neural signals in the absence of task-related demands, thereby minimizing confounding influences from active task engagement (Boly et al., 2007; Zang et al., 2007); it is worth noting that the concept of “baseline” brain activity is a contentious issue, as others have argued that no such state exists (Morcom and Fletcher, 2007; Gusnard and Raichle, 2001). Further, because resting-state does not require the extra burden of being paired with a task, between-study comparisons are more straightforward.

## 2 Inconsistent data collection parameters in resting-state NIRS

We reviewed the resting-state studies included in Butters and colleagues' publication and concluded there was considerable inconsistency in how resting-state NIRS data was gathered (Table 1; Butters et al., 2023). Specifically, researchers collected resting-state data anywhere from 60 s to 20 min, or, more troubling, the exact length of time was not indicated in nine (~28%) of the 32 studies. Further, we noted variability in the instructions provided to participants regarding eye condition. Specifically, two (~6%) studies instructed participants to keep their eyes open, while nine (~28%) told them to keep their eyes closed. One study instructed participants to alternate between eyes open/closed, and 63% (*n* = 20) did not specify, at least within the reported methods, what participants should do with their eyes. For the three studies that executed an eyes-open condition, the fixation symbol was either not specified or inconsistent between studies. Finally, we noted that the studies employed divergent nomenclature (i.e., “resting-state,” “rest period,” “baseline,” etc.) and instructions regarding what to think about (i.e., “think of nothing in particular,” “do not think about anything,” etc.). We considered whether such inconsistencies would resolve as the body of research expanded. When investigating the prevalence of these inconsistencies in studies (*n* = 19) from the last five (i.e., since 2019) years, we found four studies (~21%) did not clarify scan length, and 10 studies (~53%) did not clarify eyes open vs. closed; such values suggest that the inconsistencies persist even in more recent work.

**Table 1:** Variability in resting-state near-infrared spectroscopy (NIRS) parameters.

**First author, year**	**Length of scan (minutes)**	**Eyes condition**	**Fixation symbol if eyes opened**
Babiloni, 2014	2 eyes open and 2 eyes closed	Open and Closed	None specified
Soo Baik, 2021	*Unclear*	*Unclear*	*Unclear*
Bär, 2007	5	*Unclear*	*Unclear*
Bu, 2019	15	Closed	*NA*
Canario, 2022	11	Closed	*NA*
Chiarelli, 2021	5	Closed	*NA*
Fallgatter, 1997	*Unclear*	*Unclear*	*Unclear*
Ferdinando, 2022	5	*Unclear*	*Unclear*
Ghafoor, 2019	4	*Unclear*	*Unclear*
Greco, 2021	*Unclear*	*Unclear*	*Unclear*
Ho, 2022	1	Open	White cross on monitor
Keles, 2022	5	Closed	*NA*
Li, 2018b	11	Closed	*NA*
Li, 2022	15	*Unclear*	*Unclear*
Schwarz, 2004	*Unclear*	*Unclear*	*Unclear*
Liu, 2014	20	*Unclear*	*Unclear*
Marmarelis, 2017	5–6	*Unclear*	*Unclear*
Marmarelis, 2021	5	*Unclear*	*Unclear*
Morimoto, 2022	*Unclear*	*Unclear*	*Unclear*
Nguyen, 2019	1	Open	White cross on black background
Niu, 2019	11	Closed	*NA*
Oyama, 2018	*Unclear*	*Unclear*	*Unclear*
Tarumi, 2014	5	*Unclear*	*Unclear*
Tatsuno, 2021	*Unclear*	*Unclear*	*Unclear*
Van Beek, 2010	5	*Unclear*	*Unclear*
Van Beek, 2012	5	*Unclear*	*Unclear*
Viola, 2013	*Unclear*	Closed	*NA*
Viola, 2014	*Unclear*	*Unclear*	*Unclear*
Yang and Hong, 2021	5	*Unclear*	*Unclear*
Yang, 2022	8	Closed	*NA*
Zeller, 2019	5	Closed	*NA*
Zhang, 2022	10	*Unclear*	*Unclear*

All included studies were classified as resting-state in the 2023 review from Butters et al. (2023).

## 3 Discussion

Research in functional magnetic resonance imaging, the “gold standard for *in vivo* imaging of the brain” (Klein et al., 2022), supports that methodological details, such as scan length, eye condition, and the fixation symbol, alter study findings. Notably, functional magnetic resonance imaging and NIRS are similar in that they share hemodynamic origins and reveal brain function through neurovascular coupling, albeit by measuring hemoglobin differently; (validation) studies spanning more than a decade suggest that the techniques overlap in their activation and connectivity profiles, yet they are not identical, and this has been attributed to the amount of data collected (i.e., runs), spatial coverage, and-or brain-scalp distance of NIRS (Novi et al., 2023a; Zinos et al., 2024; Cui et al., 2011; Uchitel et al., 2022). Using functional magnetic resonance imaging, Birn and colleagues determined that longer resting-state scans (i.e., 12+ min vs. the “standard” 5–7 min) improved reliability due to an increase in both the time points and scan length (Birn et al., 2013). Weng and colleagues found the brain to be more active and less stable in an eyes-closed condition (Weng et al., 2020). Findings from Patriat and colleagues indicated there is a small but significant difference in network-specific activity when participants fixate on a cross versus a non-fixated eyes-open condition (Patriat et al., 2013). To the best of our knowledge, no systematic review has been executed on scan length, eyes open vs. closed, and/or fixation symbol, but additional work (Han et al., 2023; Agcaoglu et al., 2019; Liu et al., 2013; Zou et al., 2015) further supports the impact of such parameters on resting-state outputs. Together, the evidence from functional magnetic resonance imaging suggests that consistent methodology is the first step in meaningful data comparisons. Therefore, while no fault of Butters and colleagues, making direct comparisons between their review's resting-state studies is not just difficult but likely incorrect.

Moving forward with the budding NIRS field, researchers must standardize elements of the data pipeline where possible. Yücel and colleagues have laid the groundwork with their seminal report on best practices (Yucel et al., 2021), while others have looked to bring consistency to specific pipeline parameters (Abdalmalak et al., 2022; Pinti et al., 2018; Tsuzuki and Dan, 2014; Notte et al., 2024); notably, none have addressed the factors highlighted in this work, underscoring its novelty and importance. Despite the two techniques not being identical, functional magnetic resonance imaging could serve as inspiration for standardizing basic, fundamental NIRS resting-state data collection parameters. For example, the Canadian Dementia Imaging Protocol harmonizes imaging acquisitions to study neurodegeneration; it requires resting-state to be acquired for a specific length and, if available, using a fixation cross (Duchesne et al., 2019). To this end, we suggest that researchers use the term “resting-state” when collecting NIRS data where participants are instructed to “let your mind wander freely.” Further, researchers collecting resting-state NIRS data should consider doing so for at least 12 min, and anything less should be clearly indicated (i.e., *abbreviated* resting-state); note that *abbreviated* resting-state differs from a rest block, which is typically incorporated before and/or after a task. We recommend 12 min as it aligns with previous studies and protocols from functional magnetic resonance imaging (Birn et al., 2013; Duchesne et al., 2019; Gunter et al., 2017; Ma et al., 2024; Abdul Wahab et al., 2022). Importantly, such a recommendation is not based on the data or time points acquired (i.e., machinery); if it were, the superior temporal resolution of resting-state NIRS might allow for a shorter scan length than those completed by functional magnetic resonance imaging. Instead, the 12-min duration is grounded in the theory that the functional connectivity of these resting-state networks evolves on a slow time scale (i.e., human physiology), which may only be partially captured by scans of shorter durations.

Providing suggestions regarding eyes open or closed and the usage and type of fixation symbol is more challenging, as researchers may need to modify these parameters depending on their research question. However, if researchers have no preference, they should consider opting for an eyes-open condition, as participants are more likely to enter a drowsy or early-sleep state with their eyes closed (Allen et al., 2018). Such states affect resting-state output, given that the default mode, one of the three core networks in the triple network model (Menon, 2011), becomes decoupled during sleep (Horovitz et al., 2009). For NIRS studies where participants are instructed to fixate on a symbol, but researchers are indifferent to the type, we suggest using a white cross on a black background, given its widespread uptake in functional magnetic resonance imaging (Patriat et al., 2013; Agcaoglu et al., 2019).

We offer these suggestions as interim guidelines until a consensus group establishes definitive standards and/or original research demonstrates that scan length, eye condition, and eye fixation symbol do not significantly influence NIRS findings or their interpretations. For example, the optimal scan length is unknown until there exists a direct comparison between various durations. We also welcome alternative suggestions, as they could spark broader discussions and potentially catalyze the formation of said consensus group to define best practices in resting-state NIRS. Overall, our findings indicate significant methodological variability among resting-state NIRS. To enhance transparency and reproducibility, we urge researchers to, at a minimum, clearly report scan length and eye condition moving forward. For studies conducted with eyes open, researchers should explicitly state whether participants were instructed to fixate on a symbol and specify the type of symbol used.

In reality, all parameters that can meaningfully influence the measured signals should be meticulously recorded and reported; this includes but is certainly not limited to: clinical classification, the NIRS device, the preprocessing pipeline, and figures and visualization. In particular, incorporating additional tools to monitor and ultimately account for physiological parameters (i.e., breathing and heart rate, mean arterial pressure, etc.) may be especially important in resting-state versus task-based paradigms, given the variability that can be introduced when activity is not driven by an external stimulus. Previous work has indeed demonstrated that “systemic physiology augmented” NIRS (i.e., SPA-NIRS) impacts study findings and reflects the “gold standard” (Novi et al., 2023b; Scholkmann et al., 2022). Of course, including such external measures is not always feasible or possible, so others have suggested potential workarounds, such as temporally shifting short-channel data (Novi et al., 2023b). Again, we direct the interested reader to the seminal work from Yücel and colleagues for guidance on best practices in reporting (Yucel et al., 2021).

NIRS is an emerging neuroimaging field that is portable, less expensive, and more accessible than (functional) magnetic resonance imaging (Furlano and Nagamatsu, 2020). Resting-state NIRS provides an opportunity to understand the human brain at baseline. Until definitive guidelines are established, adopting interim standards for resting-state NIRS—specifically, collecting data for at least 12 min with participants fixating on a cross in an eyes-open condition—can help reproducibility and facilitate between-study comparisons, ultimately enhancing our understanding of the human brain in those living with and without dementia.
